# Eating behaviour, physical activity, TV exposure and sleeping habits in five year olds: a latent class analysis

**DOI:** 10.1186/s12887-021-02640-0

**Published:** 2021-04-17

**Authors:** Molly Mattsson, Deirdre M. Murray, Mairead Kiely, Fergus P. McCarthy, Elaine McCarthy, Regien Biesma, Fiona Boland

**Affiliations:** 1 Division of Population Health Sciences, Royal College of Surgeons in Ireland, Dublin, Ireland; 2 RCSI Department of Epidemiology and Public Health Medicine, Beaux Lane House, Lower Mercer Street, Dublin 2, Ireland; 3 Department of Paediatrics and Child Health, University College Cork, Cork, Ireland; 4 Cork Centre for Vitamin D and Nutrition Research, School of Food and Nutritional Sciences, University College Cork, Cork, Ireland; 5 Irish Centre for Maternal and Child Health Research, Cork University Maternity Hospital, University College Cork, Cork, Ireland; 6 University Medical Center Groningen, University of Groningen, Groningen, Netherlands; 7 Data Science Centre, Royal College of Surgeons in Ireland, Dublin, Ireland

**Keywords:** Eating behaviour, Physical activity, Childhood obesity, Latent class analysis

## Abstract

**Background:**

Diet, physical activity, sedentary behaviours, and sleep time are considered major contributory factors of the increased prevalence of childhood overweight and obesity. The aims of this study were to (1) identify behavioural clusters of 5 year old children based on lifestyle behaviours, (2) explore potential determinants of class membership, and (3) to determine if class membership was associated with body measure outcomes at 5 years of age.

**Methods:**

Data on eating behaviour, engagement in active play, TV watching, and sleep duration in 1229 5 year old children from the Cork BASELINE birth cohort study was obtained through in-person interviews with parent. Latent class analysis was used to identify behavioural clusters. Potential determinants of cluster membership were investigated using multinomial logistic regression. Associations between the identified classes and cardio metabolic body measures were examined using multivariate logistic and linear regression, with cluster membership used as the independent variable.

**Results:**

51% of children belonged to a normative class, while 28% of children were in a class characterised by high scores on food avoidance scales in combination with low enjoyment of food, and 20% experienced high scores on the food approach scales. Children in both these classes had lower conditional probabilities of engaging in active play for at least 1 hour per day and sleeping for a minimum of 10 h, and higher probability of watching TV for 2 hours or more, compared to the normative class. Low socioeconomic index (SEI) and no breastfeeding at 2 months were found to be associated with membership of the class associated with high scores on the food avoidance scale, while lower maternal education was associated with the class defined by high food approach scores. Children in the class with high scores on the food approach scales had higher fat mass index (FMI), lean mass index (LMI), and waist-to-height ratio (WtHR) compared to the normative class, and were at greater risk of overweight and obesity.

**Conclusion:**

Findings suggest that eating behaviour appeared to influence overweight and obesity risk to a greater degree than activity levels at 5 years old. Further research of how potentially obesogenic behaviours in early life track over time and influence adiposity and other cardio metabolic outcomes is crucial to inform the timing of interventions.

**Supplementary Information:**

The online version contains supplementary material available at 10.1186/s12887-021-02640-0.

## Introduction

Obesity is an important risk factor for multiple non-communicable diseases (NCD) and is associated with increased mortality [1–5]. Children and adolescents with obesity have been found to be approximately five times more likely to have obesity in adulthood compared to children without obesity [6]. Behaviours that directly or indirectly contribute to energy imbalance, including dietary patterns, physical activity (PA), sedentary behaviours, and sleep time are considered major contributory factors of the increased prevalence of childhood overweight and obesity [7]. Furthermore, these behaviours have been found to often coexist and interrelate, and to be established at an early age [7]. Identifying clusters of obesogenic behaviours may allow us to identify children at risk of overweight and obesity and to target interventions at vulnerable groups.

Exploratory data-driven methods have been increasingly used to examine patterns of lifestyle behaviours in children and adolescents [8]. Methods include cluster analysis (CA), a heuristic cluster technique which involves linking cases by looking at all possible pairs of cases and linking those in the pair with the smallest distance, then continuing in this manner until all cases lie in one large cluster, and latent class analysis (LCA), a model-based clustering approach that derives clusters using a probabilistic model [9]. A key advantage of model-based over heuristic cluster techniques is that they provide fit statistics and information on the probability that an individual is within a particular class [10].

Several literature reviews have analysed the results of studies using CA or LCA to identify lifestyle behaviour patterns in children and adolescents [8, 11, 12]. Lifestyle behaviours children and adults have been found to cluster in complex ways and differ by gender, age and socioeconomic status (SES). Findings to support an association between obesogenic cluster patterns and overweight and obesity have so far been inconclusive [8, 11]. Most previous studies have been conducted in older children, while evidence in young children (< 6 years), where children’s lifestyle behaviours are nearly entirely dependent on parental influences, is more limited. One recent study analysed behavioural patterns at 2 and 5 years of age, and identified two clusters characterised by opposite eating habits at 2 years, while TV was the most distinguishing cluster feature at 5 years. Girls in the cluster defined by high TV exposure were found to have significantly higher body fat at 5 years [13]. Another study investigating behavioural patterns in 2–9 year olds found that clusters characterised by high sedentary behaviour, low fruit and vegetable intake, sugar-sweetened beverage consumption and low PA were associated with body mass index (BMI) and waist circumference z-scores greater than 1 [14]. Moreover, few studies have studied diet, physical activity, sedentary behaviour, and sleep simultaneously, with sleep in particular often not included [15, 16]. Previous research on eating behaviour clusters or profiles is also limited, with most studies having examined the quality of diet using food frequency questionnaires (FFQ) or dietary recall [8]. The Child Eating Behaviour Questionnaire (CEBQ) is a parent-report questionnaire designed to capture individual differences in aspects of eating style that may contribute to both underweight and overweight [17]. CEBQ has previously been used to identify eating behaviour reflecting fussy/picky eating in children using latent profile analysis (LPA) [18], however it has not previously been considered in conjunction with other lifestyle behaviours.

The aims of this cross-sectional study were threefold: (1) to identify groups of children based on eating behaviour, activity-related behaviours, and sleep at 5 years of age using LCA; (2) to explore determinants of class membership, namely sociodemographic and maternal characteristics and early feeding practices, and (3) to determine if class membership was associated with body measure outcomes at 5 years of age, including overweight and obesity, fat mass index (FMI), lean mass index (LMI), and waist-to-height ratio (WHtR).

## Methods

### Study population and design

Study subjects were participants of the Cork BASELINE (Babies after SCOPE: Evaluating the Longitudinal Impact using Neurological and Nutritional Endpoints) Birth Cohort Study [19], a mother–infant prospective birth cohort study based in Cork, Ireland. It was initiated in 2008 as a follow-up to the SCOPE (Screening for Pregnancy Endpoints) Ireland study, a major multi-centre prospective pregnancy study involving primiparous low-risk women. One thousand five hundred thirty-seven SCOPE participants consented for their infants to participate in the BASELINE study and during a second stream of recruitment, a further 646 infants were recruited after delivery from the postnatal wards of Cork University Maternity Hospital, with primiparous low risk women having a singleton pregnancy being the main inclusion criterion. Paediatric follow-up with in-person assessments were conducted at birth, 2, 6, and 12 months and at 2 and 5 years. Data on the child’s early-life environment, diet, health and development were recorded at each assessment. In a subgroup of children (*n* = 591), body composition and bone mineral density (total body and lumbar spine) were measured at 5 years using dual-energy X-ray absorptiometry (DXA) (GE Healthcare Lunar iDXATM). In total, 2172 infants were recruited. One thousand two hundred twenty-nine of the children had data at the 5 year assessment and was used for the purpose of this analysis; thus representing a 43% attrition rate. To evaluate sample size requirements for analysis such as LCA, Monte Carlo data simulation techniques are frequently used [20]. While no such techniques were applied in this study, a recent study investigating sample size requirements for structural equation models identified a range of sample size requirements from 30 to 460 cases across a wide range of models [20]. Our sample of 1229 children may therefore be deemed sufficient.

### Children’s lifestyle factors included in LCA

Eating behaviour at 5 years of age was assessed using the CEBQ. The CEBQ consists of 35 items scored on a 5-point Likert scale from 1 ‘never’ to 5 ‘always’. Items are assigned to eight subscales: Emotional Overeating (EOE), Food Responsiveness (FR), Enjoyment of Food (EF), Desire to Drink (DD), Emotional Undereating (EUE), Satiety Responsiveness (SR), Food Fussiness (FF) and Slowness in Eating (SE). Subscales represent two dimension; “food approach” (EOE, EF, FR, DD) and “food avoidance” (EUE, SR, FF, SE). All items are listed in Supplementary Table 1. The mean score was calculated for each subscale and expressed as z-scores. Physical activity and TV watching were assessed from the responses to the questions “In the past month, on average, how many hours a day is your child involved in active play (such as running, jumping, climbing, sports)?” and “In the past month, on average, about how many hours a day does your child spend sitting still watching TV/videos?”. Dichotomous variables were created for active play < 1 h per day and ≥ 1 h per day and TV watching < 2 h per day and ≥ 2 h per day based on national and international guidelines and World Health Organization (WHO) recommendations on sedentary behaviour [21]. A dichotomous variable was created for sleep duration (< 10 h or ≥ 10 h) as 10–14 h sleep has been recommended by the American Academy of Sleep Medicine as appropriate for children aged 5 years [22].

### Sociodemographic and maternal characteristics and early feeding

Maternal educational attainment, SES, marital status, smoking status, activity levels and BMI were assessed at the 5 year assessment. SES was determined using the New Zealand Socioeconomic Index (SEI), an occupation-based measure of socio-economic status constructed using census data [23]. A variable was created for SEI < 24, which represents the bottom SES group in the six-group classification proposed at development of the index. Maternal participation in physical activity and TV watching were assessed from the responses to the questions “Do you participate in recreational physical activity/sport besides walking?” and “In the past month on average how many hours a day have you spent watching TV/DVD/Screen based activity (not during work hours?”. A dichotomous variable was created for TV watching with categories < 2 and ≥ 2 h per day. Maternal BMI was obtained according to standard operating procedures using digital scales and categorised as under/normal weight (BMI < 25), overweight (BMI > =25 and < 30), or obese (BMI > =30). Breastfeeding status was obtained at 2 months and categorised as any breastfeeding vs no breastfeeding. Timing of introduction of solids was assessed at 6 month and a dichotomous variable was created for introduction to solids < 18 weeks.

### Child anthropometric measurements

Weight, length, and waist circumference at 5 years were obtained according to standard operating procedures. Naked weight was measured using digital scales correct to the nearest 0.1 kg. Standing height was measured using a wall mounted stadiometer. Body composition (including fat mass and lean mass) and bone mineral density (total body and lumbar spine) was measured using DXA. DXA uses low emission x-rays to determine exact ratios of muscle, fat and bone [24]. These measurements were used to calculate WHtR, BMI, FMI, and LMI (calculated as body weight, fat, and lean mass divided by square of height). Weight status was assessed according to the International Obesity Task Force BMI cut-offs, with the cut-offs for 5.5 years of age used [25].

### Statistical analysis

Population characteristics for the total sample and latent classes were obtained using tabulation to obtain frequencies for categorical variables, while the mean and standard deviation (SD) were calculated for continuous variables.

#### Aim 1: LCA

To identify clusters of children, we conducted LCA using variables concerning eating behaviour, physical activity, sedentary behaviour, and sleep as outlined above. We used the maximum likelihood robust estimator to account for missing data by full information maximum likelihood (FIML). This process approximates missing data by estimating a likelihood function for each individual based on variables that are present, such that all the available data points are used [26]. The optimal number of latent classes was identified based on six model-fit indices: Akaike information criterion (AIC), sample-size adjusted Bayesian information criterion (BIC), adjusted Bootstrap likelihood ratio test (BLRT), Lo-Mendell Rubin test (LMRT), entropy, and interpretability of the trajectories. Lower AIC and BIC values indicate a better model fit, while the BLRT and LMRT provide a *p*-value indicating whether a model with one less trajectory group (k-1 model) should be rejected in favour of a model with k trajectories [27]. Entropy is a statistic that ranges from 0 to 1 with high values (> 0.8) indicating that individuals are classified with confidence [28]. Distinct classes were coded as a categorical variable (with k number of categories) and were named based on their visual appearance. Analysis was conducted using Mplus version 8 [29].

#### Aim 2: determinants of class membership

Associations between sociodemographic and maternal characteristics (educational attainment, socioeconomic index, marital status, smoking status, physical activity other than walking, TV watching, and weight status), child sex, and early feeding (breastfeeding at 2 months and timing of introduction to solids) and latent class membership were examined using multinomial logistic regression, with class membership the outcome of interest and the most commonly occurring class chosen as the reference category.

#### Aim 3: associations between class membership and overweight, obesity, FMI, LMI, and WHtR

Associations between class membership and overweight and obesity were examined using logistic regression, with overweight and obesity at 5 years the outcomes of interest. Associations between class membership and FMI, LMI, and WHtR were examined using linear regression, with FMI, LMI, and WHtR the outcomes of interest. These models were further adjusted for maternal characteristics, sex, and early feeding as described in section 2.3 of the Methods. Analysis was conducted using Stata version 14 [30].

## Results

Descriptive statistics of maternal (educational attainment, SEI, marital status, weight status, smoking status, physical activity, and TV watching) and child (sex, breastfeeding, introduction of solids, physical activity, TV watching, and CEBQ subscale scores) characteristics for the total sample and by latent class membership are outlined in Table 1.

**Table 1:** Population characteristics for total sample and by latent class membership

	Total (***n*** = 1229)	Class 1: Normative (***n*** = 644)	Class 2: High food avoidance (***n*** = 336)	Class 3: High food approach (***n*** = 248)
Maternal characteristics				
**Educational attainment (*n*** **= 1217)**				
No third level	94 (7.7)^a^	32 (5.0)	37 (11.2)	25 (10.1)
Some third level	371 (30.5)	186 (29.1)	111 (33.6)	74 (30.0)
Degree or higher	752 (61.8)	422 (65.9)	182 (55.2)	148 (59.9)
**Socioeconomic index < 24 (*n*** **= 1228)**				
No	1094 (89.1)	594 (92.4)	279 (83.0)	221 (88.8)
Yes	134 (10.9)	49 (7.6)	57 (17.0)	212 (11.2)
**Marital status (*n*** **= 1228)**				
Single	71 (5.8)	32 (5.0)	26 (7.7)	13 (5.2)
Married/de-facto relationship	1157 (94.2)	611 (95.0)	310 (92.3)	236 (94.8)
**Weight status (n = 984)**				
Underweight/normal weight	507 (52.5)	275 (54.0)	131 (50.2)	101 (51.5)
Overweight	292 (30.2)	145 (28.5)	86 (33.0)	61 (31.1)
Obese	167 (17.3)	89 (17.5)	44 (16.9)	34 (17.4)
**Smoking status (*n*** **= 1228)**				
No	1095 (89.2)	589 (91.6)	284 (84.5)	222 (89.2)
Yes	133 (10.8	54 (8.4)	52 (15.5)	27 (10.8)
**Physical activity other than walking (*n*** **= 1228)**	24.9 ± 4.0	24.9 ± 4.1	24.8 ± 3.9	25.3 ± 4.1
No	493 (40.2)	254 (39.5)	136 (40.5)	103 (41.4)
Yes	735 (59.9)	389 (60.5)	200 (59.5)	146 (58.6)
**Watching TV > = 2 h per day (*n*** **= 1188)**				
No	859 (72.3)	448 (72.3)	228 (70.4)	183 (75.0)
Yes	329 (27.7)	172 (27.7)	96 (29.6)	61 (25.0)
Child characteristics				
**Sex (*n*** **= 1229)**				
Male	631 (51.3)	318 (50.4)	192 (30.4)	121 (19.2)
Female	598 (48.7)	326 (54.5)	144 (24.1)	128 (21.4)
**Breastfeeding at two months (*n*** **= 1205)**				
No	601 (50.1)	340 (56.6)	142 (23.6)	119 (19.8)
Yes	604 (49.9)	294 (48.7)	183 (30.3)	127 (21.0)
**Introduction to solids < 18 weeks (*n*** **= 1193)**				
No	854 (71.6)	456 (73.2)	221 (68.0)	177 (72.2)
Yes	339 (28.3)	167 (26.8)	104 (32.0)	68 (27.8)
**Active play > =1 h per day (*n*** **= 1228)**				
No	160 (13.0)	61 (9.5)	59 (17.6)	40 (16.1)
Yes	1068 (87.0)	582 (90.5)	277 (82.4)	209 (83.9)
**Watching TV > = 2 h per day (*n*** **= 1228)**				
No	1024 (83.4)	568 (88.3)	250 (74.4)	206 (82.7)
Yes	204 (16.6)	75 (11.7)	86 (25.5)	43 (17.3)
**Sleeping > = 11 h per night (*n*** **= 1228)**				
No	633 (51.6)	289 **(**45.0)	194 **(**57.7)	150 (60.2)
Yes	595 (48.5)	354 **(**55.0)	142 **(**42.3)	99 (39.8)
**EF subscale score**^**b**^ **± SD (*n*** **= 1226)**	3.78 **±** 0.75	4.01 **±** 0.53	2.9 **±** 0.51	4.3 **±** 0.51
**EOE subscale score**^**c**^ **± SD (*n*** **= 1226)**	1.71 **±** 0.61	1.51 **±** 0.41	1.48 **±** 0.46	2.52 **±** 0.57
**DD subscale score**^**d**^ **± SD (*n*** **= 1227)**	2.56 **±** 0.90	2.31 **±** 0.76	2.74 **±** 1.01	2.97 **±** 0.87
**FF subscale score**^**e**^ **± SD (*n*** **= 1226)**	2.94 **±** 0.90	2.61 **±** 0.70	3.80 **±** 0.76	2.64 **±** 0.77
**SE subscale score**^**f**^ **± SD** **(*n*** **= 1227)**	3.08 ± 0.78	2.89 ± 0.65	3.75 ± 0.63	2.69 ± 0.71
**EUE subscale score**^**g**^ **± SD (*n*** **= 1226)**	2.44 ± 0.85	2.25 ± 0.78	2.66 ± 0.93	2.62 ± 0.82
**FR subscale score**^**h**^ **± SD (*n*** **= 1226)**	2.38 ± 0.76	2.12 ± 0.49	2.06 ± 0.54	3.47 ± 0.55
**SR subscale score**^**i**^ **± SD (*n*** **= 1226)**	2.97 ± 0.61	2.80 ± 0.46	3.58 ± 0.47	2.60 ± 0.53

^a^n (%); indicates frequency and column percentages

^b^Enjoyment of food

^c^Emotional overeating

^d^Desire to drink

^e^Food fussiness

^f^Slowness in eating

^g^Emotional undereating

^h^Food responsiveness

^j^Satiety responsiveness

### Aim 1: LCA results

**Fig. 1:**
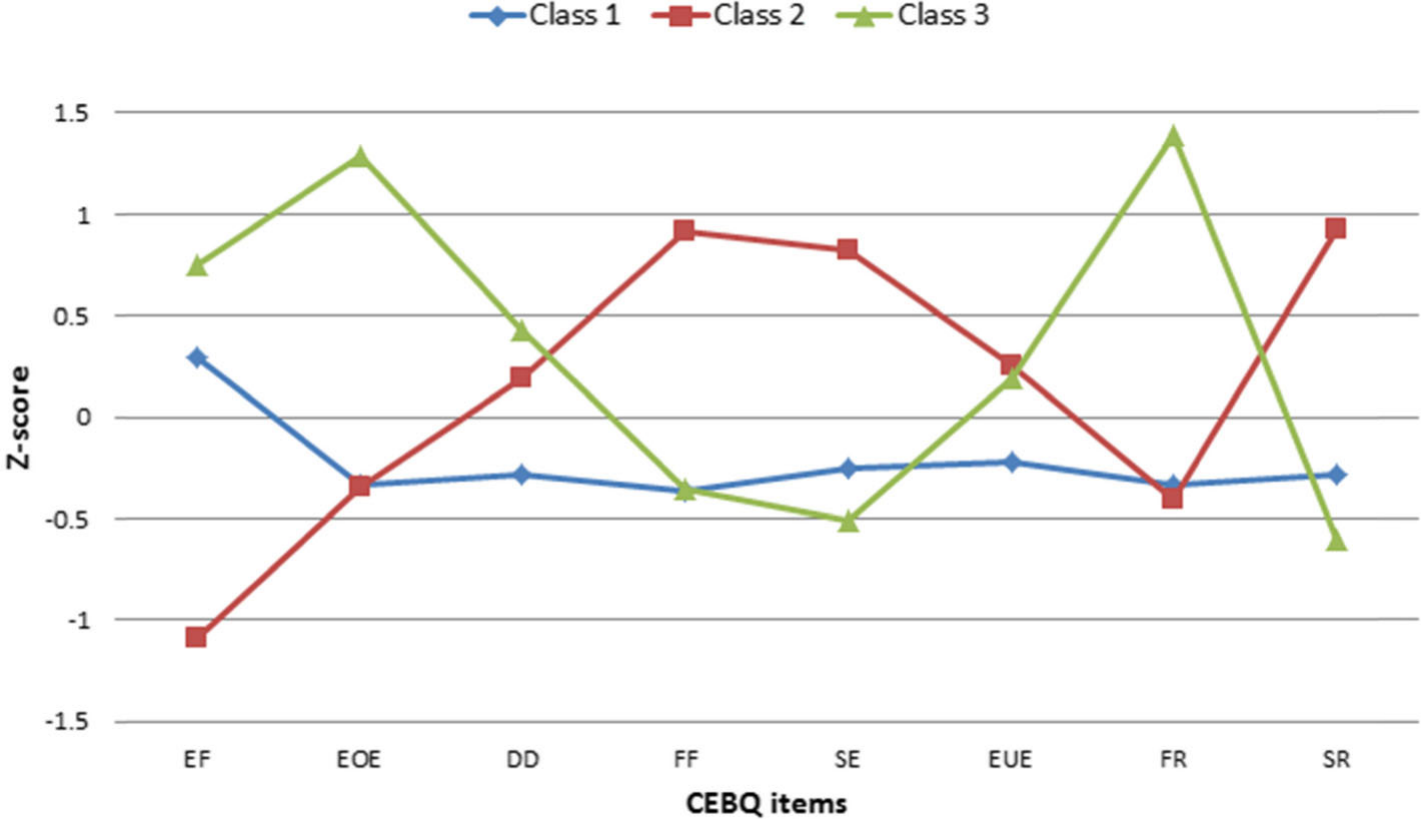
CEBQ by class. Class 1: Normative class. Class 2: High food avoidance and low enjoyment of food. Class 3: High food approach. EF = Enjoyment of food. EOE = Emotional overeating. DD = Desire to drink. FF = Food fussiness. SE = Slowness in eating. EUE = Emotional undereating. FR = Food responsiveness. SR = Satiety responsiveness

LCA of the CEBQ subscales, daily activity level, TV-watching, and sleep duration indicated three distinct classes. The three-class model was selected based on the fit indices in Table 2. AIC, BIC, and BLRT indicated a better fit for a class model, however LMRT indicated that a four class model was not significantly superior to the three class one. Additionally, entropy was slightly superior for the three-class model. To illustrate how the behaviours differ between the classes, the conditional probabilities of active play ≥1 h/day, TV-watching ≥2 h/day, and sleep duration ≥10 h/night are described in Table 3 and Fig. 1 shows the pattern of CEBQ subscale scores for each of the three identified classes. 51% of children had CEBQ subscale scores within 1SD and had higher conditional probabilities of engaging in active play for at least 1 hour per day and sleeping for a minimum of 10 h, and lower probability of watching TV for 2 hours or more, compared to the two other classes. This class may be classified as the *normative class*. Class 2 (28%) was found to be characterised by a pattern of high scores on food avoidance scales (food fussiness, slowness in eating, and satiety responsiveness) in combination with low enjoyment of food, and will be classified as the *high food avoidance class*. Class 3 (20%) experienced high scores on the food approach scales (enjoyment of food, emotional overeating and food responsiveness) and will be classified as the *high food approach class.*

**Table 2:** Fit indices

No. of classes	Class proportions	AIC^**a**^	Sample-size adjusted BIC^**b**^	LMRT^**c**^ for ***k-1*** versus k classes	BLRT^**d**^ for ***k-1*** versus ***k*** classes	Entropy
		Value	Value	***P***-value	***P***-value	
**2**	C1: 820 (66.7%) C2: 409 (33.3%)	30,350	30,410	< 0.001	< 0.001	0.78
**3**	C1: 644 (52.4%) C2: 336 (27.3%) C3: 249 (20.3%)	29,580	29,663	< 0.001	< 0.001	0.79
**4**	C1: 467 (38.0%) C2: 285 (23.2%) C3: 309 (25.1%) C4: 168 (13.7%)	29,303	29,410	0.0752	< 0.001	0.77
**5**	C1: 166 (13.5%) C2: 416 (33.8%) C3: 301 (24.4%) C4: 88 (7.2%) C5: 258 (21.0%)	29,093	29,223	0.0168	< 0.001	0.79

^a^Akaike information criterion

^b^Bayesian information criterion

^c^Bootstrap likelihood ratio test

^d^Lo-Mendell Rubin test

**Table 3:** Conditional probabilities by latent class

	Total (***n*** = 1229)	Class 1: (***n*** = 644)	Class 2: (***n*** = 336)	Class 3: (***n*** = 248)
**Active play > =1 h/day**				
No	0.13	0.09	0.18	0.16
Yes	0.87	0.91	0.83	0.84
**Watching TV > =2 h/day**				
No	0.83	0.88	0.75	0.83
Yes	0.17	0.12	0.25	0.17
**Sleeping > =10 h/night**				
No	0.52	0.45	0.57	0.60
Yes	0.49	0.55	0.43	0.40

### Aim 2: determinants of class membership

For class 2, the high food avoidance class, compared to class 1 (the normative class), SES and breastfeeding were found to be associated. SEI < 24 increased the risk of membership of this class (Relative risk ratio (RRR) (95% Confidence Interval (CI)): 1.82 (1.09–3.07)), while any breastfeeding at 2 months was found to be inversely associated (RRR (95% CI): 0.64 (0.45–0.91)). Membership of class 3, the high food approach class, was found to be associated with educational level, with attainment of a degree, compared to no third level education, inversely associated (RRR (95% CI): 0.44 (0.22–0.90)). No associations were found for mother’s weight or lifestyle behaviours, including smoking, participation in physical activity other than walking, and TV watching for 2 h or more per day. Table 4 provides a full outline of the multinomial logistic regression analysis.

**Table 4:** Multinomial logistic regression results for determinants of latent class membership

	Class 1: Normative RRR^**a**^ (95% CI^**b**^) ***p***-value	Class 2: High food avoidance RRR (95% CI) ***p***-value	Class 3: High food approach RRR (95% CI) ***p***-value
Maternal characteristics			
**Educational attainment (vs no third level)**			
Some third level	Ref	0.66 (0.35–1.24) 0.195	0.64 (0.32–1.30) 0.215
Degree or higher	Ref	0.54 (0.28–1.02) 0.057	0.44 (0.22–0.90) **0.024**
**Socioeconomic index < 24 (vs ≥ 24)**	Ref	1.82 (1.09–3.07) **0.023**	1.10 (0.59–2.06) 0.76
**Marital status (vs single)**			
Married/de-facto relationship	Ref	0.64 (0.34–1.22) 0.177	0.90 (0.42–1.91) 0.776
**Weight status (vs underweight /normal weight)**			
Overweight^c^	Ref	1.12 (0.78–1.62) 0.539	1.21 (0.82–1.80) 0.337
Obese^d^	Ref	0.74 (0.46–1.17) 0.197	0.95 (0.58–1.56) 0.837
**Current smoker (vs nonsmoker)**	Ref	1.46 (0.91–2.35) 0.12	1.15 (0.66–2.00) 0.62
**Physical activity participation (vs no participation)**	Ref	0.95 (0.68–1.33) 0.785	1.04 (0.73–1.49) 0.81
**Watching TV ≥ 2 h per day (vs < 2 h per day)**	Ref	0.96 (0.67–1.38) 0.67	0.74 (0.49–1.11) 0.58
Child characteristics			
**Child sex (vs male)**			
Female	Ref	0.80 (0.58–1.11) 0.179	1.06 (0.75–1.49) 0.761
**Breastfeeding at two months (vs not breast-feeding)**	Ref	0.64 (0.45–0.91) **0.013**	1.01 (0.69–1.46) 0.973
**Introduction to solids < 18 weeks (≥18 weeks)**	Ref	0.98 (0.68–1.42) 0.923	0.89 (0.59–1.34) 0.58

^a^Relative risk ratio

^b^Confidence interval

^c^BMI > =25 & < 30

^d^BMI > =30

### Aim 3: class membership and weight status and cardio metabolic measures at 5 years

Associations between latent class membership and body outcome measures at 5 years of age are displayed in Table 5. Class 2, the high food avoidance class, had significantly lower FMI and LMI compared to Class 1. These differences persisted after adjusting for demographic and maternal characteristics, child sex, and early feeding. Conversely, class 3, the high food approach class, had significantly higher FMI, LMI, and WtHR. Additionally, class 3 had a significantly higher risk of overweight and obesity at 5 years. Children in this class were 2.88 (95% CI 1.86–4.47) times as likely to have overweight compared children in class 1, and 5.11 (95% CI 1.81–14.41) times as likely to have obesity. No significant differences in weight status were identified between class 1 and class 2.

**Table 5:** Multivariate logistic and linear regression results for the associations between class membership and cardio metabolic measures

	Overweight^**a**^ (***n*** = 1135)^**b**^	Obesity^**c**^ (***n*** = 1135)^**b**^	FMI^**d**^ (***n*** = 591)^**b**^	LMI^**e**^ (***n*** = 591)^**b**^	WtHR^**f**^ (***n*** = 1129)^**b**^
	OR^**g**^ (95% CI^**h**^) ***p***-value	OR (95% CI) ***p***-value	β coefficient (95% CI) ***p***-value	β coefficient (95% CI) ***p***-value	β coefficient (95% CI) ***p***-value
**Class 1: Normative**	Ref.	Ref.	Ref.	Ref.	Ref.
**Class 2: High food avoidance**	0.63 (0.37–1.07) 0.088	0.66 (0.16–2.77) 0.572	−.25 (−.47, −.02) **0.033**	−.48 (−.70, −.26) **< 0.001**	−.002 (−.007, .003) 0.477
**Class 3: High food approach**	2.88 (1.86–4.47) **< 0.001**	5.11 (1.81–14.41) **0.002**	.42 (.18, .67) **0.001**	.30 (.06, .54) **0.013**	.01 (.006, .02) **< 0.001**

^a^BMI > =17.42 for boys, BMI > =17.25 for girls

^b^Adjusted for maternal characteristics (educational attainment, socioeconomic index, marital status, smoking status, physical activity other than walking, TV watching, and weight status), child sex, and early feeding (breastfeeding at 2 months and timing of introduction to solids)

^c^ BMI > =19.46 for boys, BMI > =19.36 for girls

^d^Fat mass index

^e^Lean mass index

^f^Waist-to-Height Ratio

^g^Odds ratio

^h^Confidence interval

## Discussion

Using latent class analysis, we identified distinct groups of children based on eating behaviour, physical activity, TV use, and sleep at 5 years of age. Approximately half of children belonged to a normative class, while 28% of children were in a class characterised by high scores on the food avoidance scales (food fussiness, slowness in eating, and satiety responsiveness) in combination with low enjoyment of food, and 20% experienced high scores on the food approach scales (enjoyment of food, emotional overeating and food responsiveness). Children in both these classes had lower conditional probabilities of engaging in active play for at least 1 hour per day and sleeping for a minimum of 10 h, and higher probability of watching TV for 2 hours or more, compared to the normative class. There was also some evidence that these classes were associated with lower maternal education level and SEI. There was evidence that children in class 3, the high food approach class, had higher FMI, LMI, and WtHR compared to the normative class, and were at greater risk of overweight and obesity. Conversely, children in class 2 had lower FMI and LMI compared to class 1. These differences persisted after adjusting for potential confounders.

To our knowledge this is the first study to include eating behaviour, as opposed to dietary quality, when examining clusters of lifestyle behaviours. The CEBQ was designed to assess eating styles related to obesity risk and has been shown to have a robust factor structure, high internal validity, and test–retest reliability. It has not been validated in an Irish sample, but was previously successfully validated in UK samples, samples which may be deemed similar to the one used for the purpose of this study [31]. LPA has previously been used with the CEBQ to identify an eating behaviour profile reflecting fussy/picky eating in children and to describe characteristics of fussy eaters [18, 32], however CEBQ scores have not been used to identify profiles of children at risk of overweight and obesity, nor has it been considered in conjunction with other lifestyle factors to identify obesogenic behaviour patterns.

Previous findings suggest that obesogenic behaviour patterns are complex, with a mixed high physical activity and high sedentary behaviour cluster observed most frequently, but with healthy and unhealthy patterning of all behaviours also reported [8]. Our study found no evidence of a mixed pattern in terms of physical activity and sedentary behaviour; as both classes deviating from the normative group in terms of eating behaviour had lower physical activity and higher sedentary behaviour levels. Similarly to previous studies [8], SES appeared to influence group membership, with children from lower socioeconomic backgrounds more likely to belong to the high food avoidance class, and children with lower educated parents more likely to the high food approach class.

Previous evidence in relation to an association between lifestyle behaviours and obesity outcomes has been inconsistent; some studies have found a higher prevalence of overweight/obesity in unhealthy classes while other studies found no such association [8]. This study found a strong association between class membership and overweight and obesity, with children in the high food approach group at approximately three times as high risk of overweight and five times as high risk of obesity compared to the normative group. As children in both class 2 and 3 had lower conditional probabilities of engaging in active play for at least 1 hour per day and sleeping for a minimum of 10 h, and higher probability of watching TV for 2 hours or more, compared to class 1, it appears that eating behaviour influences overweight and obesity risk to a greater degree than activity levels. Diet has previously been found to be more strongly associated with weight and weight loss than exercise and physical activity. While negative energy balance and subsequent weight loss can be achieved by either reducing energy intake or increasing energy expenditure, exercise-induced weight loss is usually small, and smaller than expected from an exercise-induced increase in energy expenditure [33, 34]. Physical activity does however have multiple benefits for adults and children alike, regardless of weight status. Previous studies have found physical activity to be inversely associated with all-cause mortality at all levels of BMI and waist circumference [35], while others have found that sustained physical activity, rather than weight loss, was associated with improved survival in coronary heart disease [36]. For children physical activity has been found to have numerous benefits. Apart from positive changes in adiposity, it is also associated with skeletal and psychological health, cognitive function and cardiorespiratory fitness [37]. Physical inactivity may therefore be an important issue to address in all groups of children, regardless of adiposity status. In our study, the children in the food avoidance class had the lowest conditional probability of engaging in active play for 60 min per day and the highest of watching TV for more than 2 hours per day. While there were no associations between membership of this class and overweight or obesity risk, it is possible that the higher levels of physical inactivity could have other implications.

Identifying how clusters of obesogenic behaviours and how they are associated with overweight and obesity may allow us to identify children at risk of overweight and obesity and to target interventions at vulnerable children and families. In this study we have found that children with high enjoyment of food, emotional overeating and high food responsiveness (including frequent requests for food and a tendency to overeat) in combination with lower activity levels were at much higher risk of experiencing overweight and obesity, compared with children in the normative class. An awareness of how eating behaviour impacts on obesity risk could potentially allow us to target appropriate interventions at children identified to be at risk at an early age, however more research is needed.

### Strengths and limitations

This study has several strengths. The relatively large sample was recruited from the only maternity hospital in Cork, Ireland, and therefore included mothers and children from a broad range of social circumstances, thus increasing the generalisability of the findings. Our study included several adiposity-specific outcomes, which were obtained by trained staff using standardised instruments using Standard Operating Procedures. Furthermore, our analytical approach is based on LCA, which involve fewer subjective decisions than clustering techniques such as CA do, and presents several advantages, specially dealing with missing values and mixed-type variables, as well as the possibility of full model selection. Another advantage of LCA is that it provides class membership probabilities for each child, which may be used to effectively assess the robustness of the classification.

However, limitations remain. Firstly, although the CEBQ is a validated tool, the study includes a reliance on parental report for all behavioural data, which is known to be vulnerable to both recall and social desirability biases. Further, measures of physical activity and sedentary behaviour were assessed using participation in active play and TV-watching as proxies, thus not providing us with a complete profile of activity-related behaviour. As the study sample consisted of predominantly Caucasian women and children (98%), ethnic differences could not be taken into account. Finally, longitudinal attrition has the potential to introduce bias. Lost to follow-up is an important issue to be considered in any cohort study due to its potential threat to study validity. Of the 2172 participants at the start of the study, 1229 were still enrolled and assessed at age five, resulting in an attrition rate of 43%. Certain methodological limitations must also be noted. The assignment of children to latent classes is based on their highest estimated group-membership probability to the identified pattern. Thus, these latent patterns should not be considered as the actual behavioural patterns but, rather, as approximations of more complex ones.

## Conclusion

We identified three latent classes based on eating behaviour, activity and sleep in children aged 5 years using a large prospective birth cohort in Ireland. Children with high food approach scores combined with lower levels of activity and sleep duration were at increased risk of overweight and obesity. Further research of how early life eating behaviour and activity levels influence overweight and obesity risk is warranted. Longitudinal evidence that examines younger children over time is vital; as understanding how the clustering of potentially obesogenic behaviours track over time and the timing of critical periods where activity levels may decline or eating behaviour change are crucial to inform the timing of interventions. Extended follow-up of cohorts is imperative in order to determine how long-term exposure of different clustering patterns is associated with overweight and obesity and other cardio metabolic measures. Additionally, more research into eating behaviours in children and how negative eating behaviours in children (e.g. emotional overeating, very frequent requests for food, tendencies to overeat) can be managed by parents and health professionals is warranted.

## Supplementary Information

**Additional file 1.**

## Data Availability

The datasets analysed during the current study are not publicly available but are available from the study principal investigator Prof Deirdre Murray on reasonable request.

## Abbreviations

NCD: Non-communicable disease
PA: Physical activity
CA: Cluster analysis
LCA: Latent class analysis
FFQ: Food frequency questionnaire
CEBQ: Child Eating Behaviour Questionnaire
LPA: Latent profile analysis
BASELINE: Babies after SCOPE: Evaluating the Longitudinal Impact using Neurological and Nutritional Endpoints
FMI: Fat mass index
LMI: Lean mass index
WHtR: Waist-to-height ratio
SCOPE: Screening for Pregnancy Endpoints
EOE: Emotional overeating
FR: Food responsiveness
EF: Enjoyment of food
DD: Desire to drink
EUE: Emotional undereating
SR: Satiety responsiveness
FF: Food fussiness
SE: Slowness in eating
WHO: World Health Organization
SES: Socioeconomic status
SEI: Socioeconomic index
BMI: Body mass index
FIML: Full information maximum likelihood
AIC: Akaike information criterion
BIC: Bayesian information criterion
BLRT: Bootstrap likelihood ratio test
LMRT: Lo-Mendell Rubin test
RRR: Relative risk ratio
CI: Confidence interval
