# Supplementary material for: Eating behaviour, physical activity, TV exposure and sleeping habits in five year olds: a latent class analysis
Source: BMC Pediatr. 2021 Apr 17;21:180. doi: 10.1186/s12887-021-02640-0 (PMC8052652; doi:10.1186/s12887-021-02640-0)
Supplement: Supplementary file 1 — Additional file 1. [file 12887_2021_2640_MOESM1_ESM.docx]

**Eating behaviour, physical activity, TV exposure and sleeping habits in five year olds: a latent class analysis**

Molly Mattsson^1*^, Deirdre M Murray^2^, Mairead Kiely^3^, Fergus P McCarthy^4^, Elaine McCarthy^4^, Regien Biesma^5#^, Fiona Boland^6#^

^1^Division of Population Health Sciences, Royal College of Surgeons in Ireland, Dublin, Ireland

^2^Department of Paediatrics and Child Health, University College Cork, Cork, Ireland

^3^Cork Centre for Vitamin D and Nutrition Research, School of Food and Nutritional Sciences, University College Cork, Cork, Ireland

^4^Irish Centre for Maternal and Child Health Research, Cork University Maternity Hospital, University College Cork, Cork, Ireland

^5^University of Groningen, University Medical Center Groningen, Groningen, Netherlands

^6^Data Science Centre, Royal College of Surgeons in Ireland, Dublin, Ireland

*Corresponding author:

Molly Mattsson

RCSI Department of Epidemiology and Public Health Medicine

Beaux Lane House, Lower Mercer Street,

Dublin 2, Ireland

mollymattsson@rcsi.ie

#Authors contributed equally

**Child Eating Behaviour Questionnaire (CEBQ)**

Please read the following statements and tick the boxes most appropriate to your child’s eating behaviour.

|  | Never | Rarely | Some-times | Often | Always |  |
| --- | --- | --- | --- | --- | --- | --- |
| My child loves food | □ | □ | □ | □ | □ | EF |
| My child eats more when worried | □ | □ | □ | □ | □ | EOE |
| My child has a big appetite | □ | □ | □ | □ | □ | SR* |
| My child finishes his/her meal quickly | □ | □ | □ | □ | □ | SE* |
| My child is interested in food | □ | □ | □ | □ | □ | EF |
| My child is always asking for a drink | □ | □ | □ | □ | □ | DD |
| My child refuses new foods at first | □ | □ | □ | □ | □ | FF |
| My child eats slowly | □ | □ | □ | □ | □ | SE |
| My child eats less when angry | □ | □ | □ | □ | □ | EUE |
| My child enjoys tasting new foods | □ | □ | □ | □ | □ | FF* |
| My child eats less when s/he is tired | □ | □ | □ | □ | □ | EUE |
| My child is always asking for food | □ | □ | □ | □ | □ | FR |
| My child eats more when annoyed | □ | □ | □ | □ | □ | EOE |
| If allowed to, my child would eat too much | □ | □ | □ | □ | □ | FR |
| My child eats more when anxious | □ | □ | □ | □ | □ | EOE |
| My child enjoys a wide variety of foods | □ | □ | □ | □ | □ | FF* |
| My child leaves food on his/her plate at the end of a meal | □ | □ | □ | □ | □ | SR |
| My child takes more than 30 minutes to finish a meal | □ | □ | □ | □ | □ | SE |

|  | Never | Rarely | Some-times | Often | Always |  |
| --- | --- | --- | --- | --- | --- | --- |
| Given the choice, my child would eat most of the time | □ | □ | □ | □ | □ | FR |
| My child looks forward to mealtimes | □ | □ | □ | □ | □ | EF |
| My child gets full before his/her meal is finished | □ | □ | □ | □ | □ | SR |
| My child enjoys eating | □ | □ | □ | □ | □ | EF |
| My child eats more when she is happy | □ | □ | □ | □ | □ | EUE |
| My child is difficult to please with meals | □ | □ | □ | □ | □ | FF |
| My child eats less when upset | □ | □ | □ | □ | □ | EUE |
| My child gets full up easily | □ | □ | □ | □ | □ | SR |
| My child eats more when s/he has nothing else to do | □ | □ | □ | □ | □ | EOE |
| Even if my child is full up s/he finds room to eat his/her favourite food | □ | □ | □ | □ | □ | FR |
| If given the chance, my child would drink continuously throughout the day | □ | □ | □ | □ | □ | DD |
| My child cannot eat a meal if s/he has had a snack just before | □ | □ | □ | □ | □ | SR |
| If given the chance, my child would always be having a drink | □ | □ | □ | □ | □ | DD |
| My child is interested in tasting food s/he hasn’t tasted before | □ | □ | □ | □ | □ | FF* |
| My child decides that s/he doesn’t like a food, even without tasting it | □ | □ | □ | □ | □ | FF |
| If given the chance, my child would always have food in his/her mouth | □ | □ | □ | □ | □ | FR |
| My child eats more and more slowly during the course of a meal | □ | □ | □ | □ | □ | SE |

**SCORING OF THE CEBQ**

**(Never=1, Rarely=2, Sometimes=3, Often=4, Always=5)**

Food responsiveness = item mean FR

Emotional over-eating = item mean EOE

Enjoyment of food = item mean EF

Desire to drink = item mean DD

Satiety responsiveness = item mean SR

Slowness in eating = item mean SE

Emotional under-eating = item mean EUE

Food fussiness = item mean FF

*Reversed items

Wardle, J, Guthrie CA, Sanderson, S and Rapoport, L. Development of the Children’s Eating Behaviour Questionnaire. *Journal of Child Psychology and Psychiatry.* **42,** 2001, 963-970.
